# Development and Validation of a Respiratory-Responsive Vocal Biomarker–Based Tool for Generalizable Detection of Respiratory Impairment: Independent Case-Control Studies in Multiple Respiratory Conditions Including Asthma, Chronic Obstructive Pulmonary Disease, and COVID-19

**DOI:** 10.2196/44410

**Published:** 2023-04-14

**Authors:** Savneet Kaur, Erik Larsen, James Harper, Bharat Purandare, Ahmet Uluer, Mohammad Adrian Hasdianda, Nikita Arun Umale, James Killeen, Edward Castillo, Sunit Jariwala

**Affiliations:** 1 Montefiore Medical Center, Albert Einstein College of Medicine Bronx, NY United States; 2 Sonde Health Boston, MA United States; 3 Deenanath Mangeshkar Hospital Pune India; 4 Brigham and Women's Hospital Boston, MA United States; 5 University of California San Diego, CA United States

**Keywords:** vocal biomarkers, COVID-19, respiratory-responsive vocal biomarker, RRVB, artificial intelligence, machine learning, asthma, smartphones, mobile phone, eHealth, mobile health, mHealth, respiratory symptom, respiratory, voice, vocal, sound, speech

## Abstract

**Background:**

Vocal biomarker–based machine learning approaches have shown promising results in the detection of various health conditions, including respiratory diseases, such as asthma.

**Objective:**

This study aimed to determine whether a respiratory-responsive vocal biomarker (RRVB) model platform initially trained on an asthma and healthy volunteer (HV) data set can differentiate patients with active COVID-19 infection from asymptomatic HVs by assessing its sensitivity, specificity, and odds ratio (OR).

**Methods:**

A logistic regression model using a weighted sum of voice acoustic features was previously trained and validated on a data set of approximately 1700 patients with a confirmed asthma diagnosis and a similar number of healthy controls. The same model has shown generalizability to patients with chronic obstructive pulmonary disease, interstitial lung disease, and cough. In this study, 497 participants (female: n=268, 53.9%; <65 years old: n=467, 94%; Marathi speakers: n=253, 50.9%; English speakers: n=223, 44.9%; Spanish speakers: n=25, 5%) were enrolled across 4 clinical sites in the United States and India and provided voice samples and symptom reports on their personal smartphones. The participants included patients who are symptomatic COVID-19 positive and negative as well as asymptomatic HVs. The RRVB model performance was assessed by comparing it with the clinical diagnosis of COVID-19 confirmed by reverse transcriptase–polymerase chain reaction.

**Results:**

The ability of the RRVB model to differentiate patients with respiratory conditions from healthy controls was previously demonstrated on validation data in asthma, chronic obstructive pulmonary disease, interstitial lung disease, and cough, with ORs of 4.3, 9.1, 3.1, and 3.9, respectively. The same RRVB model in this study in COVID-19 performed with a sensitivity of 73.2%, specificity of 62.9%, and OR of 4.64 (*P*<.001). Patients who experienced respiratory symptoms were detected more frequently than those who did not experience respiratory symptoms and completely asymptomatic patients (sensitivity: 78.4% vs 67.4% vs 68%, respectively).

**Conclusions:**

The RRVB model has shown good generalizability across respiratory conditions, geographies, and languages. Results using data set of patients with COVID-19 demonstrate its meaningful potential to serve as a prescreening tool for identifying individuals at risk for COVID-19 infection in combination with temperature and symptom reports. Although not a COVID-19 test, these results suggest that the RRVB model can encourage targeted testing. Moreover, the generalizability of this model for detecting respiratory symptoms across different linguistic and geographic contexts suggests a potential path for the development and validation of voice-based tools for broader disease surveillance and monitoring applications in the future.

## Introduction

### Background

Voice-based health assessments using acoustic signatures (“vocal biomarkers”) are well suited to be considered as prescreening and population health surveillance tools, as they do not require additional hardware beyond the smartphone itself and are therefore inherently accessible and low cost. Vocal biomarkers have already shown promise in various areas of health condition detection, including mental health [1,2], neurodegeneration [3], and cardiovascular conditions [4]. Approaches like these, using acoustic features (“how you sound”) and linguistic analysis (“what you say”), have indicated that <10 seconds of voice recording can be adequate to obtain results consistent with medical diagnoses or validated as screening instruments in approximately 60% to 90% of cases. These techniques are feasible on consumer devices operating under naturalistic conditions, outside of controlled laboratory environments [5]. Vocal biomarker applications for the detection of potential COVID-19 could be particularly interesting, as they could be used frequently and at the population scale, thereby helping to detect and monitor the spread of the disease in the population. These approaches could be complementary to polymerase chain reaction (PCR) and rapid tests, which are costlier, less accessible, and do not return results quickly [6,7]. The digital nature of the approach also has the potential to serve as a centralized monitoring system, similar to wastewater RNA detection [8], but at a lower operational cost and potentially more easily applicable to future pandemics that have a respiratory component.

Several groups have investigated voice-based identification of COVID-19 using machine learning– and artificial intelligence–based methods on a labeled data set of voice recordings from patients with COVID-19 and control groups [9-23]. Although these reports included a variety of model training and testing approaches, with different participant recruitment methods, they all used newly collected data to train models to identify COVID-19. Our work differs substantially in that we tested an existing respiratory-responsive vocal biomarker (RRVB) model, previously developed using an asthma and healthy control data set, for its ability to extend, without alteration, to a COVID-19 population.

### Objective

This study was not designed to optimize COVID-19 identification. The intent was primarily to validate the existing RRVB model on a new data set from different geographies, settings, and medical conditions. The ability to preserve performance on such diverse independent data sets is not routinely examined (or achieved) with machine learning approaches and, if successful, would support the robustness of this RRVB model. One published report that attempted to do so in a COVID-19 data set found a marked decline in detection performance on a new test set [24].

As the study was not aimed at developing a COVID-19 detection model, the enrollment criteria had several specific requirements. The most significant one was the need for patients who are COVID-19 positive (COV +ve) to have at least 1 COVID-19–associated symptom (respiratory or otherwise). This was performed to avoid a high percentage of asymptomatic cases [25], which may not be detectable with the existing RRVB model. As detailed in the *Results* section, the enrolled participants did have a mix of symptom burden, including some that were asymptomatic at the time of voice recordings, allowing an analysis of the effect of symptom burden on RRVB performance. In addition to patients who were COV +ve, patients who were symptomatic and were tested negative for COVID-19 were also enrolled. This was done to enable comparisons with other acute illnesses that have a similar symptom spectrum to COVID-19.

## Methods

### RRVB Model Development

The RRVB model was trained using data collected from over 20 hospitals in India from August 2018 to January 2020 and included 5 major Indian languages and English. This digital biobank includes over 3000 patients with respiratory disease, of whom 1700 were diagnosed with asthma, as well as a similar number of healthy controls. A more detailed description of the model development and testing is provided in Multimedia Appendix 1.

In summary, a detection model was trained using a 6-second–held “ahh” vowel elicitation and was optimized to differentiate patients with asthma from healthy controls. The model was evaluated for its ability to generalize to hold out validation data from patients with asthma, as well as individuals with other respiratory diseases including chronic obstructive pulmonary disease (COPD), interstitial lung disease (ILD), and cough. Acoustic features were selected based on univariate correlations with the 2 training classes and evaluated by repeated stratified cross-validation on several potential confounding variables (eg, phone manufacturer, research coordinator, and language). Candidate features were combined, and an exhaustive set of these feature combinations was trained using a logistic regression model using the same stratification process as described in Multimedia Appendix 1. The model performance was evaluated based on the area under the curve of the receiver operating characteristic, and the coefficients of the final logistic model were determined using the full training data set. The model produces a score ranging from 0 to 100, representing the likelihood that the modeled voice characteristics more closely resemble those of patients with asthma than those without. A threshold value (fixed at 65) was used to convert to a binary screening outcome of good health or asthma diagnosis (negative vs positive). Performance on a test set of patients with asthma, COPD, ILD, and cough indicated that performance was comparable across all these conditions: sensitivity and specificity ranged from 55% to 75%, with odds ratios (ORs) ranging from 3 to 9. The generalizability across these conditions suggests that the RRVB model responds to a subset of voice acoustic changes that accompany the shared symptoms across these conditions (ie, shortness of breath, cough, chest tightness, or pain).

### COVID-19 Study Description

The primary objective of this study was to determine whether the RRVB model developed to identify people with a medical diagnosis of asthma can also identify patients who were COV +ve from asymptomatic healthy volunteers (HVs); a third cohort of patients who were symptomatic but COVID-19 negative (COV −ve) was also enrolled. The study design and end points were prespecified as described at ClinicalTrials.gov (NCT04582331) [26].

Participating sites, in decreasing order of total participant enrollment, were Deenanath Mangeshkar Hospital, Pune, Maharashtra, India; Montefiore Medical Center, Bronx, New York; Brigham and Women’s Hospital, Boston, Massachusetts; and University of California, San Diego Health, San Diego, California.

The study targeted patients at presentation to the clinical site (typically emergency department or urgent care), although admitted patients were also eligible to enroll for up to 5 days after testing (see the *Eligibility Criteria* section). Patients with COVID-19–like symptoms could enroll before a confirmed COVID-19 diagnosis, and the study design intended both positive and negative patients to continue with the study. Diagnosis for participants in the COV −ve group was not collected. HVs were recruited from hospital staff or their family members and were required to be asymptomatic with no history of positive COVID-19 tests (viral or serological).

**Figure 1:**
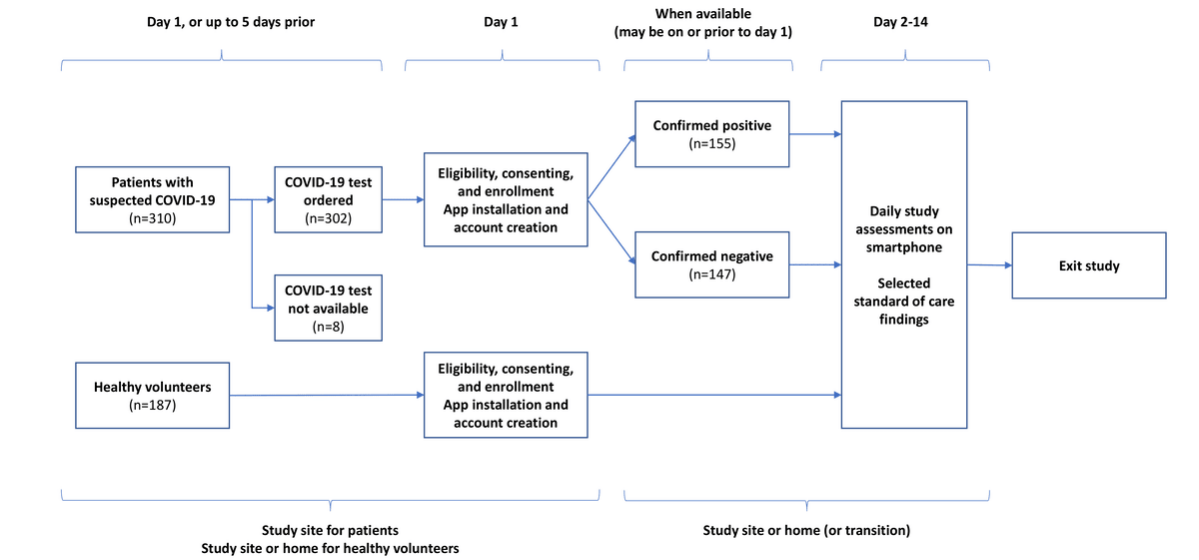
Participant journey: participants were identified when presenting with suspected COVID-19. Testing and any additional standard practice assessments (not shown) confirmed either positive or negative clinical diagnosis status, and all patients continued through daily study assessments through day 14 to exit of study. Healthy volunteers follow a similar journey, without COVID-19 testing. Daily assessments are conducted on the participants’ personal smartphone devices using the study app. Actual enrollment numbers are shown.

Figure 1 graphically illustrates the participant journey. Upon agreeing to participate, participants were instructed to download the study app (described in the *Mobile App* section) onto their personal smartphone, provide in-app electronic consent, and record daily voice samples and symptom inventories for 14 days (day 1-14), without further involvement of the study team beyond the day 1 baseline session (see the *Assessments and Data Collection* section for more details). Participants displayed varying levels of compliance with the daily study tasks, but the primary end point used day-1 data only, which all the participants provided.

Most participants were enrolled in person, although a small number were enrolled virtually while at home (if they were previously seen and tested at the study site). In-app study assessments were completed by participants at their treatment location for as long as they remained there, with instructions to continue at home if they were not admitted or discharged before day 14.

### End Points

The primary end points were the sensitivity, specificity, and OR of the RRVB model screening result (explained in the *RRVB Screening* section) to identify patients who were COV +ve versus HVs on day 1. The primary end point was evaluated for the entire participant group, as well as for various subgroups defined by demographic, geographic, or clinical factors. The same set of analyses was conducted for COV –ve group versus HVs on day 1.

### Eligibility Criteria

Both patients who were COV +ve and COV −ve presenting with at least one of the following COVID-19–associated symptoms, for up to 10 days before enrollment on day 1—cough, fever (>37.5 °C or 99.5 °F), shortness of breath, sore throat, diarrhea, anosmia, or ageusia—were eligible to participate, provided a COVID-19 test was performed no more than 5 days before day 1.

Patients with critical disease were not eligible, as the intended uses of this technology are most likely for early identification of disease or monitoring of patients in nonacute settings. Critical disease was defined as patients on supplemental oxygen via a nonrebreather mask or high-flow nasal cannula, presenting with one or more of hypoxemic respiratory failure or acute respiratory distress syndrome on continuous positive airway pressure or bilevel positive airway pressure or invasive ventilation, shock, multiorgan dysfunction, or multisystem inflammatory syndrome in children. HVs were not eligible if they had a history of a positive COVID-19 viral or serological test result at any time before enrollment, but no test was ordered as part of this study.

The participants were required to own a smartphone that could download and run the study app and sign up for a user account. Participants were required to be able to speak and read either English or Spanish (US sites) or Marathi or English (India site).

### Mobile App

The study app was previously developed by Sonde Health to conduct vocal biomarker studies and is an integrated component of a broader vocal biomarker platform that includes cloud-based back-end services and databases, as well as web-based configuration tools to support different study designs. For research studies, the application was configured to obtain electronic informed consent, questionnaire responses, and voice sample recordings. The app can be used with iOS (version 12.4.4, iPhone 6 or later; Apple Corp) or Android devices (operating version 7.0 or later).

The app configuration was achieved via a token that was specific to each site and the desired language of the participant (English, Spanish, or Marathi). Language choice allowed participants to read informed consent, questionnaires, and voice sample elicitation instructions in their preferred language.

At enrollment, participants were trained by study coordinators on the proper use of the app, in particular requirements for good quality voice sample recordings (being in a quiet place with minimal background noise and providing the correct type of voice elicitation).

### Assessments and Data Collection

Participant assessments (except for COVID-19 testing and standard of care clinical tests) were conducted on the study app and involved audio (voice) recordings (without facial mask) of a 6-second–held vowel “ahh” as in the word “father” (other elicitations were recorded but not analyzed as part of this report) and a symptom questionnaire. The questionnaire included a 12-item symptom inventory previously used in a large web-based study of COVID-19 symptoms [27], which is consistent with the Centers for Disease Control and Prevention (CDC) coronavirus guidance, and one additional question regarding the participant’s location at the time of the study session (outpatient clinic, inpatient, home, or other location). The symptom question responses were generally yes or no, with a few that had a subjective severity scale. The symptoms included were fever; persistent cough; unusual fatigue; unusual shortness of breath or trouble breathing; sore or painful throat; loss of smell or taste; unusually hoarse voice; unusual chest pain or tightness; skipping meals; gastrointestinal symptoms (diarrhea, vomiting, and abdominal pain); eye infection; and rash or sores involving the mouth, hands, or feet.

During the first study session, participants also responded within the app to a one-time onboarding questionnaire, which included the participants’ ethnicity, race, duration of current symptoms, symptoms experienced at enrollment, number of days since coming to the hospital, location from where the participant came (home or nursing home), preexisting health conditions (asthma, COPD, congestive heart failure, diabetes, and hypertension), use of supplemental oxygen at home on a regular basis (yes or no), and smoking behavior (both tobacco and vaping).

Clinical data collected at each site included: brand and model of COVID-19 PCR test kit, result and date of test, and laboratory diagnosis (COV +ve or COV –ve); vital signs during the 12 hours before enrollment (highest body temperature, highest heart rate, lowest systolic blood pressure, highest respiratory rate, and lowest SpO_2_); disease severity at enrollment, classified as severe (one or more of dyspnea, hypoxia, or >50% lung involvement on imaging within 24-48 hours, including any patient on supplemental oxygen via nasal cannula) or mild (not severe or critical, including patients with no or mild pneumonia without supplemental oxygen) and any increase through the hospital stay; respiratory support requirement (room air or nasal cannula with highest flow rate); imaging results, if available; and mortality. Imaging results were investigated for the presence of opacities or infiltrates that were not fully explained by effusions, lobar or lung collapse, nodules, cardiac failure, or fluid overload and marked as present or not present. Adverse events possibly related to the study, as well as enrollment in other COVID-19 therapeutic trials, were also tracked.

### Voice Sample Processing

Voice samples recorded on participant smartphones were securely transmitted and stored on a cloud server using Health Insurance Portability and Accountability Act–compliant architecture as wav files at 44.1 kHz and down sampled to 16 kHz before further processing. Each file is then checked for quality using an “Elicitation Check” (ELCK) algorithm, scored with the RRVB model, and assigned a positive or negative result based on the comparison to a threshold (fixed at 65). The steps are explained in the following sections.

### ELCK Algorithm

Meaningful RRVB model scores required that the recorded audio was compliant with the required elicitation, which was 6-second–held “ahh” vowel (as in the word “father”), at constant volume, pitch, and tone quality. Sonde Health developed a proprietary algorithm called ELCK based on existing data sets separate from this study to automatically reject voice samples that do not sufficiently adhere to the intended elicitation. This algorithm could not be applied in real time during data acquisition and was therefore applied retroactively to the recorded audio. Samples deemed noncompliant according to the ELCK algorithm were excluded from the per-protocol analysis. Live product deployments included ELCK during voice sample collection so that analyzed recordings would meet quality criteria, minimizing the likelihood of processing and scoring less-reliable voice samples. This would also reduce the rate of sample rejection, as users would be prompted to repeat the voice sampling if necessary.

### RRVB Scoring

Voice samples were subsequently analyzed to produce RRVB scores, which ranged from 0 to 100, with higher scores indicating similarity of the participant’s selected vocal feature values to those of people diagnosed with asthma. The RRVB scores analyzed in this study were produced by first calculating predetermined acoustic feature values required as inputs to the model from the audio files and then feeding them into the RRVB model. Details regarding the original RRVB model data set, model building, and testing (separate from the COVID-19 study) are provided in Multimedia Appendix 1.

### RRVB Screening

The RRVB model scores were converted to binary results to facilitate screening use cases. This was achieved by assigning positive or negative results to the RRVB model scores relative to an established threshold (fixed at 65). Specifically, scores below the threshold were considered negative, whereas scores at or above the threshold were considered positive (Multimedia Appendix 1).

### Statistical Considerations

#### General Methodology

The data collected in this study were documented using summary tables. Categorical variables were summarized as frequencies and percentages. *P* values were calculated for ORs using a 2-sided likelihood ratio test. A .05 significance level was used, unless noted otherwise. No adjustments for covariates or multiple comparisons were applied.

#### Handling of Dropouts and Missing Data

Participants who failed to continue conducting the app study sessions were considered dropouts. This did not affect the primary end point analysis, which was based on day-1 data only, which all the participants provided. HVs, by definition, were considered negative for fever (vital signs were not obtained for HVs). Therefore, in the analysis regarding fever status, all numbers (true positive, true negative, false positive, and false negative) had 0.5 added to the calculation of the OR. This adjustment approach was used in other subgroup analyses that would otherwise lead to a noncalculable OR. No other imputation for the missing data was performed.

#### Analysis Population

The per-protocol analysis cohort of participants was defined based on the following criteria:

1. Participants other than HVs who did not have a confirmed COVID-19 diagnosis status were excluded from the analysis set.
2. ELCK result: any voice samples that failed to meet the quality criteria according to the ELCK algorithm (described in the *ELCK Algorithm* section) were excluded from the analysis.

### Statistical Analyses

The primary endpoints were sensitivity, specificity, and OR of RRVB screening results versus enrollment group as ground truth labels (COV +ve, COV −ve, or HVs). Sensitivity and specificity were summarized using the estimate and a 95% Wilson binomial CI for each proportion. The OR was summarized using the estimate and the 95% likelihood ratio CI. The null hypothesis that the true OR equals 1 was tested using a 2-sided likelihood ratio test. The OR was calculated as the odds of positive RRVB model screening results versus negative RRVB model screening results for the positive class divided by the analogous odds for the negative class. The positive class would typically be either COV +ve or COV −ve, whereas the negative class would be HVs, unless otherwise noted. The interpretation of these end points (detection metrics) for different definitions of the positive and negative classes is further addressed in the *Discussion* section.

The primary analysis was repeated separately by geographic region to determine whether vocal analysis was equally effective at different geographic locations. As sample sizes permitted, the analyses of sensitivity, specificity, and OR were also performed separately by demographic, symptom, and clinical factors.

Various additional analyses were performed. The sensitivity, specificity, and OR of COVID-19 screening based on temperature alone (presence of fever: highest temperature in the 12 hours before enrollment was >99.5 °F [>37.5 °C]) were measured orally for each participant. The US CDC states a cutoff of 100.4 °F (38 °C) for fever screening in COVID-19, but we chose to use the lower value for increased screening sensitivity, similar to others [28]. The second examined the sensitivity, specificity, and OR of COVID-19 screening based on RRVB model screening results and temperature. A positive screen required a temperature of >99.5 °F (>37.5 °C) or a positive RRVB model screening result (or both). The primary analysis was also repeated separately by symptom status (asymptomatic, no respiratory but other symptoms, and respiratory symptoms) to determine whether vocal analysis was equally effective in patients who experienced respiratory symptoms and those who did not. Finally, the primary analysis was conducted separately based on disease severity and the presence or absence of objective imaging findings.

### Ethics Approval

All research protocols were reviewed and approved before study initiation by the following ethics committees or institutional review boards: Deenanath Mangeshkar Hospital (IHR_2020_Jul_BP_375), Montefiore Medical Center (IRB# 2020-11934, reference #066166), Brigham and Women's Hospital (IRB 2020P002840), and University of California, San Diego (201718X). In addition, all participants provided electronic informed consent in the study app before participating in the study, as approved by each institution’s review board. This study was conducted in accordance with the principles of good clinical practice. Study data collected through the study app were deidentified, as participants were identified through their participant ID only; each participating site maintained a separate linking table to enable approved data from medical records to be entered into the study data set, but no personally identifiable information was included. Participants created accounts on the study app by using their email or mobile phone number; these credentials were stored in physically and functionally separate servers and were not accessible to the research staff. The study app and databases were hosted on Amazon Web Services Cloud servers. Participants received incentives in the form of electronic gift vouchers (US sites, US $25) or cash compensation (India site, INR 500 [US $6.1]) upon enrollment in the study.

## Results

### Enrollment, Demographics, and Clinical Presentation

A total of 497 participants were enrolled (Table 1) across the 4 study locations (COV +ve: n=155, 31.2%; COV −ve: n=147, 29.6%; HVs: n=187, 37.6%; without COVID-19 test results: n=8, 1.6%) between September 10, 2020, and April 28, 2021.

The per-protocol analysis cohort examining responses from study day 1 included 283 participants (COV +ve: n=97, 34.3%; COV −ve: n=70, 24.7%; and HVs: n=116, 41%). Participants (214/497, 43.1%) not included in the per-protocol analysis cohort were excluded for the following reasons:

1. COVID-19 tests were not ordered, or the results were not available (8/214, 3.7%), and
2. The participants failed voice sample quality control algorithm ELCK (206/214, 96.3%).

The large number of excluded participants owing to failing the ELCK is further addressed in the *Discussion* section.

Demographic and clinical characteristics for the analysis cohort of the 3 subgroups are provided in Table 2 and can be summarized in that the HV subgroup had a higher representation of female, mostly Asian and White individuals, balanced between the 2 geographies. The COV +ve and −ve subgroups had relatively more male individuals and a more diverse racial and language background, with a higher number of participants from India versus the United States.

Clinically, the COV +ve subgroup appeared sicker than the COV −ve subgroup, both in terms of disease severity and the presence of findings on imaging (see the *Methods* section). Vital signs in the period up to 12 hours before enrollment trended worse for the COV +ve group, although these differences were modest.

Patient-reported factors trended in the same direction, with a higher prevalence of all symptom types in COV +ve than in COV −ve group. Most patients who were COV +ve (59/97, 61%) reported 1 or more respiratory symptoms on the day of enrollment, which was markedly higher than that for COV −ve (31/70, 44%), and the reporting of any symptom was 86% (83/97) and 71% (50/70), respectively. These symptom reports were derived from the study onboarding questionnaire, separately from symptom reports made during voice sample recording on day 1, which were reported later.

The onset of symptoms and arrival at the hospital indicated that the COV +ve subgroup had generally been ill longer than the COV −ve group on day 1. The overall rate of preexisting health conditions was higher in COV +ve than in COV −ve group (41/97, 42%, vs 23/70, 33%). Asthma, COPD, and congestive heart failure were more prevalent in COV −ve group, whereas hypertension and diabetes were more prevalent in COV +ve. The HV group had a much lower prevalence of preexisting health conditions (15/116, 12.9%) than the other groups. Asthma diagnosis was correlated with the enrollment group, being 2 to 3 times more prevalent in COV +ve and COV −ve groups than in HVs, but low enough in actual prevalence (11.3% and 17.1%) that this would not likely be a significant confounder.

Stratification factors that are not shown in Table 2 were mostly answered similarly by both the COV +ve and COV −ve groups: nearly all came from home to the hospital, did not use supplemental oxygen at home on a routine basis, and indicated that they had never smoked or vaped.

**Table 1:** Patient disposition.

				HVs^a^, n (%)		COV +ve^b^, n (%)		COV –ve^c^, n (%)		Without any results, n (%)
**Overall**										
	Enrolled patients (n=497)		187 (37.6)		155 (31.2)		147 (29.6)		8 (1.6)	
	Per-protocol analysis cohort (n=283)		116 (41)		97 (34.3)		70 (24.7)		0 (0)	
	**The United States**									
		Enrolled patients (n=198)	88 (44.4)		54 (27.3)		50 (25.3)		6 (3)	
		Per-protocol analysis cohort (n=124)	65 (52.4)		35 (28.2)		24 (19.4)		0 (0)	
	**India**									
		Enrolled patients (n=299)	99 (33.1)		101 (33.8)		97 (32.4)		2 (0.7)	
		Per-protocol analysis cohort (n=159)	51 (32.1)		62 (39)		46 (28.9)		0 (0)	

^a^HV: healthy volunteer.

^b^COV +ve: COVID-19 positive.

^c^COV –ve: COVID-19 negative.

**Table 2:** Demographics and clinical presentation.

Variable				HV^a^ (n=116)		COV +ve^b^ (n=97)		COV –ve^c^ (n=70)
**Demographics, n (%)**								
	**Sex**							
		Male	41 (35.3)		58 (60)		32 (46)	
		Female	75 (64.7)		39 (40)		38 (54)	
	**Age category (years)**							
		≥18 to <65	113 (97.4)		86 (89)		66 (94)	
		≥65	3 (2.6)		11 (11)		4 (6)	
	**Language**							
		English	78 (67.2)		32 (33)		26 (37)	
		Spanish	0 (0)		11 (11)		4 (6)	
		Marathi	38 (32.8)		54 (56)		40 (57)	
	**Are you Hispanic or Latino?**							
		Yes	8 (6.9)		18 (19)		13 (19)	
		No	108 (93.1)		79 (81)		57 (81)	
	**Race**							
		American Indian or Alaska Native	1 (0.9)		1 (1)		2 (3)	
		Asian	65 (56)		64 (66)		44 (63)	
		Black or African American	9 (7.8)		5 (5)		10 (14)	
		Native Hawaiian or Other Pacific Islander	0 (0)		0 (0)		0 (0)	
		White	34 (29.3)		9 (9)		5 (7)	
		Other	6 (5.2)		14 (14)		8 (11)	
		Multiracial	1 (0.9)		4 (4)		1 (1)	
	**Geographic Region**							
		The United States	65 (56)		35 (36)		24 (34)	
		India	51 (44)		62 (64)		46 (66)	
**Clinical findings (medical chart)**								
	**Disease severity, n (%)**							
		N/A^d^	116 (100)		2 (2)		2 (3)	
		Mild	0 (0)		79 (81)		63 (90)	
		Severe	0 (0)		16 (17)		5 (7)	
	**Opacities or infiltrates on imaging, n (%)**							
		N/A (no imaging performed or result not available)	116 (100)		62 (64)		47 (67)	
		Not present	0 (0)		14 (14)		18 (26)	
		Present	0 (0)		21 (22)		5 (7)	
	**Vital signs (averages per group, 12-hour period before enrollment), mean**							
		Highest body temperature (Fahrenheit)	N/A		99		99	
		Highest heart rate (bpm)	N/A		91		87	
		Lowest systolic blood pressure (mm Hg)	N/A		123		125	
		Highest respiratory rate (bpm)	N/A		20		20	
		Lowest SpO_2_	N/A		97		98	
**Patient-reported information (day 1), n (%)**								
	**Which symptoms are you experiencing today?^e^**							
		Cough	0 (0)		47 (48)		21 (30)	
		Fever	0 (0)		42 (43)		24 (34)	
		Shortness of breath	0 (0)		24 (25)		14 (20)	
		Sore throat	0 (0)		20 (21)		7 (10)	
		Diarrhea	0 (0)		10 (10)		7 (10)	
		Loss of smell	0 (0)		15 (16)		4 (6)	
		Loss of taste	0 (0)		19 (20)		5 (7)	
		Any respiratory symptoms (cough, shortness of breath, and sore throat)	0 (0)		59 (61)		31 (44)	
		Any symptom (≥1)	0 (0)		83 (86)		50 (71)	
		I have no symptoms	116 (100)		14 (14)		20 (29)	
	**How many days ago did you first come to the hospital?**							
		Today	0 (0)		30 (31)		31 (44)	
		Yesterday	0 (0)		17 (18)		10 (14)	
		2-3 days ago	0 (0)		17 (18)		13 (19)	
		4-5 days ago	0 (0)		15 (16)		2 (3)	
		>5 days ago	1 (0)		17 (18)		5 (7)	
		N/A (HV)	115 (98.8)		1 (1)		9 (13)	
	**How many days ago did you first experience symptoms of your current illness?**							
		Today	1 (0.9)		2 (2)		7 (10)	
		Yesterday	0 (0)		5 (5)		13 (19)	
		2-3 days ago	0 (0)		26 (27)		21 (30)	
		4-5 days ago	0 (0)		17 (18)		5 (7)	
		More than 5 days ago	1 (0.9)		47 (49)		7 (10)	
		I have no symptoms	114 (98.3)		0 (0)		17 (24)	
	**Do you currently have any of the following health conditions?^e^**							
		Asthma	7 (6)		11 (11)		12 (17)	
		COPD^f^	1 (0.9)		1 (1)		2 (3)	
		CHF^g^	0 (0)		0 (0)		4 (6)	
		Diabetes	3 (2.6)		17 (18)		9 (13)	
		Hypertension (high blood pressure)	6 (5.2)		29 (30)		12 (17)	
		None of the above	101 (87)		56 (58)		47 (67)	
		Any of the above	15 (12.9)		41 (42)		23 (33)	

^a^HV: healthy volunteer.

^b^COV +ve: COVID-19 positive.

^c^COV –ve: COVID-19 negative.

^d^N/A: not applicable.

^e^More than 1 category may apply; therefore, the percentages may sum to >100%.

^f^COPD: chronic obstructive pulmonary disease.

^g^CHF: congestive heart failure.

### Primary End Point Results

For the primary end point (Table 3), discrimination performance for COV +ve group versus HV, the model exhibited 73.2% sensitivity, 62.9% specificity, and OR 4.64 (95% CI 2.58-8.33; *P*<.001). Discrimination between COV −ve group and HVs resulted in 58.6% sensitivity, 62.9% specificity, and OR 2.40 (*P*=.005).

Performance according to the primary end points by subgroup (Table 4; COV +ve vs HVs only) indicated better performance for female participants than for male participants. Despite displaying a lower sensitivity among male participants, the specificity was approximately 20% higher. However, the difference in the OR (5.42 vs 2.99) was not statistically significant (*P*=.33). For the US study sites, the sensitivity was 77.1%, specificity was 53.8%, OR was 3.94 (*P*=.004), whereas for samples collected in India, the sensitivity was 71%, specificity was 74.5%, and the OR was 7.15 (*P*<.001). Age group analysis indicated a similar sensitivity >65 and <65 years of age (81.8% vs 72.1%, respectively), but the number of participants in the older age group was relatively small, especially in the HV cohort. Subgroups according to ethnicity, race, and preferred language were also reported; however, no statistically significant differences were observed.

**Table 3:** Primary end points.

			Estimate (%; 95% CI)		*P* value
**COVID-19 positive vs healthy volunteers**					<.001
	Sensitivity	73.2 (63.6-81.0)			
	Specificity	62.9 (53.9-71.2)			
	Diagnostic odds ratio	4.64 (2.58-8.33)			
**COVID-19 negative vs healthy volunteers**					.005
	Sensitivity	58.6 (46.9-69.4)			
	Specificity	62.9 (53.9-71.2)			
	Diagnostic odds ratio	2.4 (1.31-4.40)			

**Table 4:** Primary end points: demographic breakdown.

		Sensitivity, estimate (%; 95% CI)	Specificity, estimate (%; 95% CI)	Diagnostic odds ratio (%; 95% CI)	*P* value
Overall		73.2 (63.6-81.0)	62.9 (53.9-71.2)	4.64 (2.58-8.33)	<.001
**Region**					
	The United States	77.1 (61.0-87.9)	53.8 (41.9-65.4)	3.94 (1.56-9.95)	.004
	India	71 (58.7-80.8)	74.5 (61.1-84.5)	7.15 (3.10-16.47)	<.001
**Sex**					
	Male	75.9 (63.5-85.0)	48.8 (34.3-63.5)	2.99 (1.27-7.06)	.012
	Female	69.2 (53.6-81.4)	70.7 (59.6-79.8)	5.42 (2.33-12.59)	.001
**Age (years)**					
	≥18 to <65	72.1 (61.8-80.5)	62.8 (53.6-71.2)	4.37 (2.38-8.01)	<.001
	≥65	81.8 (52.3-94.9)	66.7 (20.8-93.9)	9 (0.52-155.25)	.13
**Ethnicity**					
	Hispanic or Latino	72.2 (49.1-87.5)	62.5 (30.6-86.3)	4.33 (0.74-25.30)	.10
	Not Hispanic or Latino	73.4 (62.8-81.9)	63 (53.6-71.5)	4.7 (2.49-9.85)	<.001
**Race**					
	American Indian or Alaska Native	75 (19.8-97.3)	25 (2.7-80.2)	1 (0.01-92.43)	>.99
	Asian	71.9 (59.9-81.4)	70.8 (58.8-80.4)	6.19 (2.88-13.27)	<.001
	Black or African American	80 (37.6-96.4)	33.3 (12.1-64.6)	2 (0.15-26.74)	.60
	Native Hawaiian or Other Pacific Islander	N/A^a^	N/A	N/A	N/A
	White	66.7 (35.4-87.9)	58.8 (42.2-73.6)	2.86 (0.61-13.40)	.18
	Other	78.6 (52.4-92.4)	66.7 (30.0-90.3)	7.33 (0.88-61.33)	.07
	Multiracial	70 (29.9-92.7)	25 (2.7-80.2)	0.78 (0.02-32.37)	>.99
**Language**					
	English	81.3 (64.7-91.1)	56.4 (45.4-66.9)	5.61 (2.08-15.15)	<.001
	Spanish	54.2 (28.6-77.7)	50 (5.5-94.5)	1.18 (0.02-69.98)	.93
	Marathi	72.2 (59.1-82.4)	76.3 (60.8-87.0)	8.38 (3.22-21.79)	<.001
**Preexisting conditions**					
	None	75 (62.3-84.5)	63.4 (53.6-72.1)	5.19 (2.51-10.74)	<.001
	Asthma	63.6 (35.4-84.8)	67.1 (25.0-84.2)	2.33 (0.34-16.18)	.39
	Diabetes	76.5 (52.7-90.4)	66.7 (20.8-93.9)	6.5 (0.46-91.93)	.17
	Hypertension	79.3 (61.6-90.2)	66.7 (30.0-90.3)	7.67 (1.12-52.32)	.04
	One or more of asthma, COPD^b^, CHF^c^, diabetes, or hypertension	70.7 (55.5-82.4)	60 (35.7-80.2)	3.63 (1.06-12.44)	.04

^a^N/A: not applicable.

^b^COPD: chronic obstructive pulmonary disease.

^c^CHF: congestive heart failure.

### Impact of Clinical Factors

Disease severity did not affect RRVB sensitivity in the COV +ve subgroup: 73.4% (n=79, mild disease) versus 75% (n=16, severe disease), but it did in the COV −ve subgroup: 55.6% (n=63) versus 100% (n=5), respectively. Although the high performance in patients with severe disease in the COV −ve subgroup has a wide CI owing to the small sample size, it is helpful to realize that these are the only patients in the COV −ve group for whom respiratory complications are highly likely. Although diagnostic information in this group was not captured, the definition of severe disease implies respiratory involvement (see the *Methods* section). Therefore, the RRVB would be expected to identify those patients effectively, whereas COV −ve with mild disease likely include conditions with and without respiratory involvement. The effectiveness of RRVB detection in such a heterogeneous group would be expected to be reduced. The absence of a marked difference in the COV +ve group could be explained by the fact that COVID-19 is a respiratory disease and that regardless of the disease severity, respiratory involvement is present in all patients. With this line of reasoning, the severity analysis outcomes suggest that the RRVB detection ability is best when the patient’s condition includes a respiratory component.

To explore whether RRVB correlated with other objective clinical findings, the outcomes were compared with the results of imaging. For the 20.5% (58/283) of participants with imaging results in the analysis set, a nonsignificant relationship was found between positive findings and positive RRVB (OR 1.42; *P*=.50). There is no expectation that a perfect relationship exists, as the pathophysiology of the imaging findings and positive RRVB outcomes are not necessarily the same.

Imaging findings alone were associated with COVID-19 status (58/283, 20.5%; OR 5.40; *P*=.006). The combination of findings on imaging and a positive RRVB outcome had a nonsignificant trend with a positive COVID-19 diagnosis (n=40; OR 3.41; *P*=.08), whereas the combination of no findings on imaging and a negative RRVB had a strong association with a negative COVID-19 diagnosis (n=18; OR 16.00; *P*=.03). The latter combination of negative RRVB and negative imaging results would be useful in ruling out COVID-19.

The performance for the subset of COV +ve and HV participants with risk factors for COVID-19 owing to one or more of the 5 preexisting conditions (n=56; 70.7% sensitivity; 60% specificity; OR 3.63; *P*=.04) was slightly lower than that of the subgroup of participants with no risk factors (n=157; 75% sensitivity; 63.4% specificity; OR 5.19; *P*<.001). The same pattern was observed for COV −ve versus HVs in these 2 subgroups (n=38; 56.5% sensitivity; OR 1.95 vs n=148; 59.6% sensitivity; OR 2.55, respectively; specificity is same as previously mentioned). The fact that preexisting conditions did not meaningfully alter RRVB detection performance appears to rule out a major confounding effect of the higher rate of preexisting conditions (including asthma and the data set on which the RRVB model was trained) in the COV +ve and COV −ve groups versus HVs.

### Impact of Symptom Status

Although the inclusion criteria of the study require COV +ve and COV −ve to have at least 1 COVID-19 associated symptom at baseline to be eligible for the study, not all patients had respiratory-related symptoms. An analysis was conducted to assess sensitivity in participants who reported one or more of the predefined respiratory symptoms (trouble breathing, cough, sore throat, or hoarse voice) versus those who did not (“respiratory-asymptomatic”) at the time of voice recording on day 1.

The sensitivity in the respiratory-symptomatic COV +ve group (n=51) was 78.4% versus 67.4% in the respiratory-asymptomatic group (n=46). For COV −ve, the sensitivity was 55.6% (n=27) versus 60.5% (n=43), respectively. Because the RRVB model was developed based on identifying people with a diagnosis of asthma, it is to be anticipated that the detection performance is enhanced in those people experiencing respiratory symptoms compared with those who are not. The findings in the asymptomatic group suggest that RRVB risk scores may be able to detect objective clinical findings that are not (yet) perceived by patients. As a comparator, the lowest oxygen saturation values in these patients in the 12-hour period before enrollment were normal (respiratory-symptomatic group mean SpO_2_ 96.5% versus 97.1% in the asymptomatic group).

Extending this line of analysis beyond respiratory symptoms, 25 COV +ve and 29 COV −ve participants reported no symptoms during voice recording on day 1 and can therefore be categorized as completely asymptomatic. For these participants, the sensitivity was 68% and 48.3%, respectively. For participants who reported between 1 and 3 symptoms (of any kind), the sensitivity was 71.8% (n=39) and 72% (n=25), whereas for participants who reported ≥4 symptoms, the sensitivity was 78.8% (n=33) and 56.3% (n=16), respectively. Although not a preplanned analysis, this incidental finding gives a range of approximately 65% to 80% sensitivity of the RRVB in COVID-19 for a range of symptom burdens.

### Combined Approaches: RRVB, Temperature Screening, and Symptoms

RRVB sensitivity to COVID-19 compared favorably with temperature, the predominant quantitative symptom measure used for screening in locations where social distancing options are limited. With a cutoff of >99.5 °F (>37.5 °C), only 15.6% (15/96) of patients who were COV +ve and 14.7% (10/68) of patients who were COV −ve were identified. Using a cutoff temperature of >100.4 °F (>38 °C) led to the identification of 8.8% (8/96) of patients with COVID-19. Combining temperature with RRVB score (either providing a positive outcome) provided 78.1% sensitivity, 62.9% specificity, OR 6.06 (*P*<.001) for COV +ve group versus HVs and 64.7% sensitivity, 62.9% specificity, OR 3.11 (*P*<.001) for COV −ve versus HVs. The substantial increase in sensitivity with the use of the RRVB demonstrates a potentially significant public health benefit compared with temperature alone.

Because of the enrollment criteria, classification based on the presence or absence of self-reported symptoms on day 1 might be expected to yield perfect performance (100% sensitivity and specificity). However, some participants who were COV +ve and −ve reported no symptoms during voice recording on day 1 and would be misclassified using such a method. Likewise, some HV did in fact report symptoms and would be misclassified as well.

Classification using self-reported symptoms for the COV +ve group versus HVs yielded 74.2% sensitivity, 90.5% specificity, and OR 27.5 (*P*<.001), whereas the COV −ve group versus HVs yielded 58.6% sensitivity, 90.5% specificity, and OR 13.5 (*P*<.001). Note that these numbers differ from what might be expected based on Table 2, but the symptom reports were based on the onboarding survey (not at the time of voice recording). Given the study design, the sensitivity was surprisingly low for both groups and provided a useful comparator to the RRVB sensitivity.

As in the analysis with fever, the RRVB results could be combined with self-reported symptoms to enhance the overall detection performance. Here, we assessed various basic options in patients who were COV +ve versus HVs. In contrast to fever, which has low sensitivity, the prevalence of nonfever symptoms is relatively high. Combining these measures (>1 of any symptom or positive RRVB) yielded 91.8% sensitivity, 55.2% specificity, and OR 13.7 (*P*<.001). Applications that require high specificity may benefit from an approach that requires both a positive RRVB and >1 symptom to be reported, which has 55.7% sensitivity, 98.3% specificity, and OR 71.6 (*P*<.001). The very high specificity would lend itself well to population screening approaches where a high positive predictive value is required, despite the lower sensitivity of this approach (Tables 5-7).

**Table 5:** Number of patients for each diagnosed respiratory condition and healthy controls used in model construction and validation.

Respiratory condition	Female, n (%)	Male, n (%)
Asthma (n=1694)	910 (53.72)	784 (46.28)
COPD^a^ (n=625)	193 (30.9)	432 (69.1)
Persistent cough (n=814)	415 (51)	399 (49)
Interstitial lung disease (n=98)	56 (57)	42 (43)
None (healthy controls; n=1705)	1012 (59.35)	693 (40.64)

^a^COPD: chronic obstructive pulmonary disease.

**Table 6:** Number of patients and healthy volunteers split by model building versus validation.

Sex		Asthma, n	Healthy, n
**Female**			
	Train	601	681
	Validate	309	331
**Male**			
	Train	517	467
	Validate	267	226

**Table 7:** Performance measures of the respiratory-responsive vocal biomarker model score with cutoff to produce binary outcome, applied across a range of respiratory conditions (combined for females and males).

Condition and statistics		Estimate (95% CI)
Sensitivity (%)		66 (62-70)
Specificity (%)		69 (65-73)
OR^a^		4.3 (3.4-5.5)
**COPD^b^ (n=625)**		
	Sensitivity (%)	77 (74-80)
	Specificity (%)	73 (69-77)
	OR	9.1 (7.0-11.8)
**Persistent cough (n=814)**		
	Sensitivity (%)	55 (52-58)
	Specificity (%)	72 (68-76)
	OR	3.1 (2.5-4.0)
**Interstitial lung disease (n=98)**		
	Sensitivity (%)	65 (55-74)
	Specificity (%)	68 (64-72)
	OR	3.9 (2.5-6.2)

^a^OR: odds ratio.

^b^COPD: chronic obstructive pulmonary disease.

## Discussion

### RRVB Detection Performance in COVID-19 Data Set

The results of our study demonstrated the generalizability of an asthma-derived RRVB to patients who were COV +ve. The performance characteristics (sensitivity, 73.2%; specificity, 62.9%; OR 4.64; *P*<.001) were similar to those obtained in the original model development and validation for asthma, COPD, ILD, and persistent cough (OR 3.1-9.1). These results are noteworthy, as COVID-19 represents a different respiratory condition than that was used to train the model, suggesting that the model is generally responsive to a common set of physiological changes across these and potentially other respiratory diseases. In addition, the study was conducted in geographies and languages beyond those used for the original model development. Age, sex, race and ethnicity, geography, and language did not alter the performance characteristics in a statistically meaningful way. We believe that the use of a simple and universal elicitation (“ahh”) is a major factor in this generalizability, as the sound is ubiquitous across most major languages. Limiting the RRVB model to a relatively small number of voice acoustic features during development and testing also likely contributes to the selection of the most robust features associated with the condition of interest and avoiding overfitting on the training data.

Although our RRVB model results in COVID-19 appear less impressive compared with the high 90% range claimed by some other reports [11,19], it remains to be seen whether the model performance would be maintained when applied to independent data sets without recalibration. Indeed, one study investigating generalization across test sets showed poor ability to do so [24]. Moreover, the intent of this study was not to optimize performance for COVID-19 detection by training on a COVID-19–specific data set but rather to test whether the existing model was generalizable to COVID-19 without modification. It should be clear that the use of this detection capability is not as a confirmatory COVID-19 test. Negative results may not ensure the absence of infection, and positive results should be subjected to further clinical evaluation.

The inclusion of a COV −ve group in this study, enrolled using the same criteria as the COV +ve group, provides several insights. Although diagnoses were not available for this group (COVID-19 was ruled out by a negative PCR test), they likely represent a mix of acute illnesses, including respiratory and nonrespiratory conditions. Sensitivity in this group overall was considerably lower than that of COV +ve group (58.6% vs 73.2%), which would be anticipated if this group included a subset of patients without respiratory involvement, consistent with the lower prevalence of self-reported symptoms that include respiratory complaints. In fact, because it is not known which participants in this group have a respiratory condition and which do not, the assignment of true or false positives and true or false negatives cannot be referenced to the actual diagnosis. Out of convenience, we therefore consider all participants in the COV −ve subgroup as positives to permit calculation of the performance metrics. The performance so calculated in this group is lower than that in COV +ve group, thereby being consistent with the intended functioning of the RRVB. For the 5 participants in the COV −ve group who were classified as having severe disease, respiratory involvement was highly likely, and indeed, the RRVB identified all 5 participants.

### Symptom Status Versus RRVB Detection Ability

We found that RRVB sensitivity increased in proportion to symptom burden, especially for COV +ve: 78.4% in patients reporting at least 1 respiratory symptom versus 67.4% in those who did not. The number of symptoms (of any kind) also affected the performance. This was an incidental finding in a post hoc analysis, as by design, all patients were intended to have at least 1 COVID-19–associated symptom. Nonetheless, some patients appeared asymptomatic by self-report at the time of voice recording on day 1, and these were still detected by the RRVB model, albeit at a 10% to 15% lower sensitivity than that for symptomatic patients. The decrease in sensitivity is to be expected, as the vocal biomarker pattern used in the detection of respiratory impairment is likely weaker or absent in asymptomatic individuals. The correlation between symptom presence or severity and RRVB outputs suggests potential applications in incorporating vocal biomarker outputs into predictive models for clinical outcomes for COVID-19 [29] or long-term health monitoring of individuals (eg, remote patient monitoring, chronic disease management, etc), but would require clinical studies that pair voice recordings with other clinical assessments over an extended period.

The correlation between RRVB scores and respiratory symptoms noted could lead to higher scores in groups with a higher prevalence of underlying respiratory conditions, such as the COV +ve and COV −ve groups as opposed to the HV group (asthma prevalence: 11.3%, 17.1%, and 6%, respectively). As noted in the *Results* section, subgroup analysis of participants without any underlying conditions showed similar performance characteristics to the overall group and the subgroup of participants with >1 underlying conditions (OR 5.19 vs 4.64 vs 3.63, nonsignificant differences), indicating that our results cannot be explained by the differences in the prevalence of underlying respiratory conditions.

The symptom-based classification of COV +ve (or −ve) group versus HVs had relatively low performance, especially considering that the study design aimed to have all patients present with at least 1 symptom and HVs with no symptoms. For the COV +ve group, this approach yielded a sensitivity of 74.2%, which was essentially no better than that of the RRVB approach (73.2%). For the COV −ve group versus HVs, the situation was the same, with a sensitivity of 58.6% for both the symptom and RRVB approaches. Comparison with fever shows an even greater difference, as only about 15% of patients who were COV +ve and COV −ve were identified using a threshold of 99.5 °F (37.5 °C). Thus, the RRVB detection approach outperforms fever and any single symptom and is on par with the use of all COVID-19–associated symptoms recommended by the CDC. One potential way to combine RRVB results with self-reported symptoms is to combine them, and it requires both to be positive to obtain an overall positive screening result.

**Figure 2:**
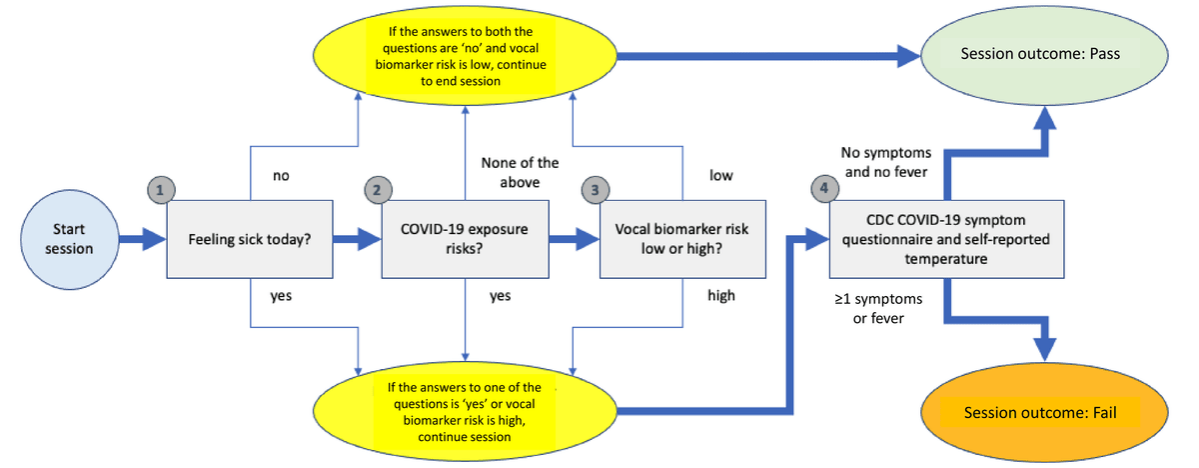
Illustrative digital triaging tool to identify people who are more likely to test positive for COVID-19 (or other acute conditions with respiratory involvement) with 4 main steps. Voice sample recording and respiratory-responsive vocal biomarker analysis occur in step 3 and determine whether a symptom survey is required (step 4). A negative respiratory-responsive vocal biomarker screening outcome bypasses the survey and produces an overall pass on screening, whereas a positive respiratory-responsive vocal biomarker screening outcome followed by at least 1 symptom being reported will result in a screen failure (positive respiratory-responsive vocal biomarker followed by no symptoms reported will produce a screen pass). Additional steps can be added to address risks that are not otherwise captured, for example, COVID-19 exposure risks (step 2) and the ability to bypass the respiratory-responsive vocal biomarker triaging step by indicating that the person knows they are sick (step 1). CDC: Centers for Disease Control and Prevention.

A simple flowchart, as shown in Figure 2, illustrates how this could be implemented in a digital triaging tool that first obtains a voice sample for RRVB analysis and continues with a CDC-recommended symptom questionnaire only if the RRVB screening result is positive (improving the user experience by not requiring a symptom survey each time). The final screening result is then determined by reporting at least 1 symptom. Additional elements could be added regarding COVID-19 exposure risks and the ability to bypass voice triaging if the user indicates feeling sick at the outset. On the basis of the results of this study, such a tool would detect patients who were COV +ve versus healthy individuals with a sensitivity of 55.7% and specificity of 98.3%. In applications where this level of sensitivity is adequate, it would provide a scalable and low-cost triaging method that would identify individuals for further follow-up with a high positive predictive value (eg, assuming a COVID-19 prevalence of 5%, positive predictive value would be 63%). Although this tool would likely also identify individuals with infectious diseases other than COVID-19, this is a benefit rather than a drawback because such individuals may also need clinical care and could also pose a risk of disease transmission. These ideas could be tested with a confirmatory study that screens the general population for early detection of COVID-19.

### Interpretation of Detection Ability

Although the RRVB model is not intended to provide a disease diagnosis, we have evaluated its performance using conventional diagnostic measures such as sensitivity (true positive rate), specificity (true negative rate), and OR. The ground truth labels necessary to calculate these measures were simply defined by the enrollment groups: patients who were COV +ve are defined as the positive class and HVs as the negative class (similarly when analyzing COV −ve vs HVs). Because the RRVB do not “detect” COVID-19, calculating performance in this way introduces some confounders. For example, participants with preexisting conditions such as asthma could be construed as a positive class regardless of the enrollment group because the RRVB model was trained to detect people with asthma. Including the preexisting conditions into the class definition would alter the assignment of participants and potentially result in different sensitivity and specificity. Likewise, the presence or absence of respiratory involvement (whether from objective clinical findings such as imaging or self-reported symptoms) could similarly be used to determine the class assignments, yielding yet another potentially different performance level. Ultimately, the “correct” definition of class and the resulting sensitivity, specificity, and OR should be based on the intended use.

### Limitations

The findings presented here should be interpreted with several limitations. First, a relatively large number of participants were excluded from the per-protocol analysis cohort because their voice sample recordings failed the automated ELCK quality control (206/497, 41.4%; *Methods* section). The ELCK was designed to eliminate less-reliable scores if the voice elicitation did not sufficiently adhere to the instructions for use. Indeed, conducting the primary end point analysis of COV +ve group versus HVs with the inclusion of voice samples that failed ELCK (n=341) reduced the performance (sensitivity, 70.3%; specificity, 58.6%; OR 3.35; *P*<.001). In contrast, for COV −ve versus HV analysis, the sensitivity improved to 67.3% (n=333). Additional examination revealed that the RRVB model scores for samples that failed ELCK were, on average, 3 to 4 points higher for COV +ve group and HVs compared with samples that passed ELCK, whereas for COV −ve, the average score increase was much larger (8.6). An exhaustive analysis of the effect of ELCK on the RRVB performance is unlikely to provide much additional insights. Limiting the analysis presented here to those samples that pass ELCK produces modest changes in the observed performance but is likely a better reflection of the underlying RRVB model performance.

RRVB-based products deployed in real-world scenarios require solutions such as the ELCK to ensure reliable elicitation scoring. These solutions can include recording additional samples if the first attempt fails. For instance, an ongoing unpublished study using the same RRVB platform in cystic fibrosis patients requested a new voice sample if ELCK failed, up to 3 per session. Of the 3975 voice samples, the ELCK success rates on the first, second, and third attempts were 95.9%, 70.1%, and 34.8%, respectively. Only <1% of the sessions remained unsuccessful after 3 attempts, and offering the second and third attempt enabled approximately 80% of the failed initial attempts to receive a usable output. Although the effectiveness of the approach may vary based on the population and setting, it appears reasonable to expect benefits in most scenarios. The higher rate of ELCK rejection in this study may reflect a more challenging acoustic environment (background noise and other talkers) in a hospital setting.

Because we have demonstrated the extension of an asthma-derived RRVB model to COVID-19 without loss of detection ability, it can be expected that future variants of COVID-19 will also be detectable with this model, but perhaps not at the same level of performance. The data for this study were collected primarily from September 2020 to December 2020, before the emergence of variants, and patients were therefore infected with the original strain of SARS-CoV-2. The first variant (alpha) appeared to have a similar prevalence of respiratory symptoms as the original strain [30,31], whereas later variants presented a different symptom prevalence in large population studies [32]. As this study has indicated a relationship between detection sensitivity and the presence and severity of symptoms (both respiratory and overall), COVID-19 variants with a higher prevalence or severity of symptoms could be expected to lead to higher detection sensitivity and vice versa. On the basis of our findings in patients who were asymptomatic versus symptomatic, a range of approximately 65% to 80% sensitivity may be reasonable for anticipating future COVID-19 variants (whereas specificity is defined based on healthy people and would not be affected by the characteristics of variants).

The performance described in this report cannot be directly extended to applications in which the patients or user population is different. Performance is determined not only by how well the vocal biomarker patterns match those that were used to train the RRVB model but also by the prevalence and severity of those vocal biomarker patterns in the test population. For example, when applying the current RRVB model to the screening of a presumptive healthy population for the early detection of (asymptomatic) COVID-19, it could be anticipated that individuals who are COV +ve include a larger fraction of patients who are asymptomatic or mildly sick than was the case in this study. Performance in this application may, therefore, be different because of the observed impact of disease severity and symptom burden on the detection performance. This would have to be tested in a use-case study that incorporates the intended use into the study design, for example, using a triaging tool, as described previously and in Figure 2. Furthermore, pediatric patients were not tested and would require additional studies.

Another limitation is that the specificity of the RRVB model has not been tested within a broader population context in which many more chronic and acute conditions are present. Although the model was trained to differentiate an asthma population from healthy controls and has been shown to be generalized across other respiratory conditions (COPD, ILD, cough, and COVID-19), it has not been tested with nonrespiratory conditions, and it is possible that its detection ability has a component that simply distinguishes sick from healthy individuals. The reported specificity of 62.9% is to be understood in the context of the negative class consisting of solely HVs, although here we defined “healthy” as not having an acute condition with COVID-19–like symptoms. It did not rule out other preexisting chronic conditions, which were present in 12.9% of HV cases (asthma, COPD, diabetes, and hypertension were included in the study onboarding survey). To understand the specificity of the RRVB model for respiratory conditions alone, the model can be tested on other nonrespiratory conditions.

As the results of this study have indicated, COV +ve and COV −ve statuses can both lead to a positive RRVB output. An analysis that would mix these groups in a detection task versus HVs would find that individuals in both groups will be detected. However, the RRVB output provides no way to help identify whether the patient is more likely to have a COV +ve or COV −ve result. Therefore, its utility is not in identifying a patient as having COVID-19 but in indicating which patients are more likely to test positive for COVID-19, captured numerically by the OR. During a pandemic, such capabilities may be useful, as they would allow identification of a subset of people from a larger population as being at high risk; these individuals could then receive appropriate follow-up examinations to obtain a definitive diagnosis. Some portions of positive RRVB outputs will be found to be completely healthy (false positive), whereas others will be found to be negative for COVID-19 but positive for another acute condition that the RRVB model detected based on their respiratory vocal biomarkers. Whether the latter case should be identified as a false positive or true positive depends on the goals of the overall screening process.

Finally, a recent paper has highlighted some general concerns with vocal biomarker–based approaches for the detection of COVID-19 [33]. Most of the concerns were related to the level of control over the participant populations, environmental conditions during voice sample recording, validity of ground truth labels, and potential confounders from non–COVID-associated attributes between groups. These were generally well controlled in this study (*Methods* section). Uniquely, the results presented on this study were derived from an existing model that was developed from a completely independent data set in a different patient population, which avoids overfitting to this particular COVID-19 data set or COVID-19–specific confounders. An evaluation that remains to be done is to determine the level of specificity of the RRVB model with respect to other conditions, including nonrespiratory conditions, as highlighted earlier. Currently, we have demonstrated a general capability to identify patients across several conditions that all have respiratory impairment as a common symptom, similar to how a thermometer generally identifies immune system activation.

The analogy with thermometers is worth further consideration. Thermometers are, by design, not specific to a particular disease, yet they have arguably been one of the most widely distributed and valuable medical devices ever invented. From the foregoing statement, it may also be clear that our RRVB model does not differentiate between COVID-19 and other diseases with respiratory symptoms. By design, it detects a subset of voice acoustic changes that generally occur across multiple pulmonary conditions, and its intended uses may include serving as an aid in identifying individuals more likely to test positive for COVID-19 and potentially for other infectious and chronic diseases. This risk stratification and monitoring can serve as a valuable public health tool in a variety of settings. Rather than seeing the lack of diagnostic specificity as a limitation, we believe that generalizability is a valuable feature. A well-validated general “respiratory thermometer” function compatible with smartphones and other voice-enabled devices could be a key enabler to increasing care access and timelines for respiratory illnesses in a broad range of health care resource settings. When this thermometer-like risk stratification function is used appropriately for early patient identification, the data and experience gained by validating a single RRVB model across a growing number of use cases can potentially catalyze its accelerated development and adoption. Although voice alone may never provide a definitive diagnosis for COVID-19, successful deployment of general RRVB technology across multiple disease categories is a path forward to generating the required data sets, at sufficient scale and diversity, necessary to gain confidence for widespread vocal biomarker technology adoption.

### Future Work

The general ability of the RRVB model to detect respiratory conditions could lead to various screening and monitoring applications. As explored in this report, one such application might be the early detection of potential COVID-19. This work would need to be extended in several ways to enable such capability.

First, a study replicating the intended use within a more general population would establish what performance could be expected for this application. Although this study has demonstrated the basic ability to detect patients who are COV +ve versus HVs, a prospective cohort study that recruits participants from a representative population would demonstrate its application in the real world.

Refining approaches to reject voice recordings that do not meet specifications in real time would be necessary to ensure that all or most uses will result in reliable RRVB model outputs. We demonstrated the use of an ELCK algorithm for this purpose, but it was subsequently applied and led to the elimination of many otherwise eligible voice recordings. More broadly, the entire tool needs to be easily understood and used properly without requiring considerable training or assistance to be effective as a population screening method. Recording proper voice elicitations in a suitably quiet environment is one of the more challenging requirements for this type of technology.

Finally, the RRVB model would not likely be the only component in a digital COVID-19–triaging tool, and it should be combined with other metrics such as patient-reported symptoms and temperature screening; an example of such a triaging tool is provided in Figure 2. Although we have presented the performance for simple combinations of these measures, more advanced combinations that use patterns of symptoms in combination with fever and RRVB may provide superior detection abilities. Data from this study could be used in the training of such an approach and then validated using newly collected data from future studies.

### Conclusions

The vocal biomarker approach for the identification of patients with asthma and healthy controls was successfully generalized to the detection of patients presenting to hospitals with COVID-19. Validating this machine learning model on a newly collected data set in different disease conditions provides confidence that robust vocal biomarker applications can be developed. We found that the detection performance in COVID-19 was influenced by self-reported symptom burden, suggesting that monitoring applications for conditions with fluctuations in respiratory function may also be possible. Vocal biomarker identification was superior in the detection of COVID-19 compared with temperature screening (fever) and comparable with self-reported symptoms according to the CDC guidelines. Combining vocal biomarker detection, self-reported symptoms, and fever status could provide valuable public health monitoring capabilities during pandemics. Although RRVB detection has a lower per-specimen sensitivity than molecular testing, it does not require any supplies, kits, or resources other than the user’s own smartphone. Testing is self-directed, without the need for skilled technicians, and the results are immediately available to the user. These factors together represent a unique profile relative to conventional testing approaches.

## Appendix

Multimedia Appendix 1 Asthma data set and overall approach.

## Abbreviations

CDC: Centers for Disease Control and Prevention
COPD: chronic obstructive pulmonary disease
COV +ve: COVID-19 positive
COV −ve: COVID-19 negative
ELCK: Elicitation Check
HV: healthy volunteer
ILD: interstitial lung disease
OR: odds ratio
PCR: polymerase chain reaction
RRVB: respiratory-responsive vocal biomarker

## Data Availability

The data sets analyzed during this study are available from Sonde Health (by e-mailing study@sondehealth.com) on reasonable request and execution of a template data use agreement between Sonde Health and the requesting party.
