# Supplementary material for: Development and Validation of a Respiratory-Responsive Vocal Biomarker–Based Tool for Generalizable Detection of Respiratory Impairment: Independent Case-Control Studies in Multiple Respiratory Conditions Including Asthma, Chronic Obstructive Pulmonary Disease, and COVID-19
Source: J Med Internet Res. 2023 Apr 14;25:e44410. doi: 10.2196/44410 (PMC10131712; doi:10.2196/44410)
Supplement: Multimedia Appendix 1 [file jmir_v25i1e44410_app1.pdf]

Appendix A

Asthma data set and overall approach

Between August 30, 2018, and January 31, 2020 Sonde Health collected vocal biomarker data from over 50,000 volunteers during outpatient visits to over 20 hospitals in India. All research subjects provided informed consent to a clinical research coordinator at the site of their outpatient visit and were then guided through a series of 4-6 vocal tasks and questionnaires while their responses and voice samples were recorded directly on a smartphone. Vocal responses were recorded primarily in the following languages: Hindi, Marathi, Telugu, Malayalam, Kannada and English. Additional demographic and health condition diagnoses metadata were collected by the clinical research coordinator from the medical charts at the time of the visit and entered into a separate system. These data were then transferred and stored securely on a remote server for processing.

From this digital biobank, a subset of data comprising over 3,000 respiratory disease patients, including approximately 1,700 with asthma were extracted and used in the training and validation of respiratory models according to the following approach. Additional samples from patients with other respiratory conditions were extracted for validation purposes.

First, sessions from all subjects with a confirmed diagnosis of asthma, Chronic Obstructive Pulmonary Disease (COPD), Interstitial Lung Disease (ILD), and persistent cough were selected along with a control set comprising healthy volunteers with no disease diagnosis. Any samples with insufficient adherence to the instructions for use due either to collection in a noisy environment or failure to hold the voice elicitation as instructed were removed. With the non-adherent/ low-quality samples excluded the number of remaining recordings available for model development and validation are summarized in Table 5.

The strategy employed for building the detection model was to train using the single condition represented by the largest number of possible samples, in this case asthma, based on the observation that accuracy and generalizability of vocal biomarkers tend to increase as a function of the amount of data available. The model developed and optimized for differentiating patients with asthma from healthy controls was then evaluated and validated for its ability to generalize to hold-out validation data of patients with asthma and healthy individuals, as well as additional samples from individuals with the other respiratory diseases. The hypothesis is that this model will perform in these other conditions based on the shared set of symptoms across many respiratory diseases, namely difficulty breathing, tightness in the chest, or persistent cough. Based on experience from other conditions, training models specific to gender has provided superior accuracy, so that approach was used in the development of respiratory models. The number of asthma subjects and healthy volunteers split by gender and training vs. validation data sets is shown in Table 6.

Model development

Given training labels associated with a health condition (asthma), voice acoustic features from the voice samples described in the previous section were examined for indication of correlation with that label. For the present application of detecting respiratory conditions, a patient holds an “ahhhh” vowel sound (as in the word “father”) for six seconds while the audio is recorded by the smartphone. After that recording is translated into an array of extracted acoustic feature representations (e.g., filter banks, cepstral analysis, and local discriminant bases, as used for a range of applications in speech and audio processing), a small subset of these that have been determined to correlate strongly with the presence or absence of respiratory disease diagnoses are then passed as inputs to the trained machine learning model.

Feature selection and all downstream models were conducted separately for each gender. The feature selection process is designed to reduce the set of features to the following stage while also reducing the risks of overfitting. The process is further designed to eliminate spurious correlations between a feature and the response variable that arises due to correlation with an exogenous variable, such as device-specific performance or biased population samples across study sites.

The labeled data (asthma vs. healthy control) is segmented into a training set (80%) and test set (20%), stratified by individual mobile device. Features were evaluated on the training set by repeated stratified cross-validation as follows:

a. 5-fold cross validation (unstratified);
b. 5-fold cross validation, stratified by phone device manufacturer;
c. 5-fold cross validation, stratified by clinical research coordinator;
d. 4-fold cross validation stratified by subject primary language (studies were conducted in 4 primary languages)

In each case, the response variable was balanced across folds. Univariate correlations were evaluated across the 19 data splits. To ensure robust performance, features were ranked by the lower bound of the 90% confidence interval for the correlation evaluated across folds (“feature robustness score”). Highly correlated features are further filtered by selecting the one with the highest robustness score.

Given the ranked list of features, an exhaustive enumeration of all feature combinations of up to 5 features was performed among the top 20 ranked features. For each feature combination, a logistic regression model was trained using the same stratification process above. For example, in the cross validation stratified by device manufacturer, a logistic model was trained on 4 folds and its performance was evaluated on the fifth fold by computing the area under the receiver-operator characteristic curve (AUC ROC). The process was repeated for each of the five folds and iterated over each stratification for a total of 19 folds. Mean and variance of the AUC ROC were then evaluated and ranked by the same robustness procedure as above. The full training data was then used to determine the coefficients for the logistic models.

A standard logistic regression architecture was used to map the weighted sum of selected speech features to a sigmoidal activation function on a 0-1 output range (reference labels 0: healthy, 1: asthma diagnosis). A binary cross-entropy loss function was used paired with a gradient descent optimization method.

Selecting among the various logistic models requires balancing model score, complexity, and the physiological interpretation of participating features. Top performing models were therefore evaluated by a speech scientist, with preference given to models with fewer features. Finally, performance of the selected models for males and for females was evaluated on the test set.

Model performance

The model performance was evaluated across multiple data segmentation groups to ensure performance across the data and reduce the possibility of over-fitting. The final features and models were selected among the models that generalized well across all segmentation groups.

The composite score produced by the model was multiplied by 100 to provide an output range 0-100 and corresponds to the likelihood of the presence of a lower respiratory tract condition. Gender-specific thresholds were chosen to provide approximately equal sensitivity and specificity according to the ROCs (male: 36, female: 55). To allow a uniform interpretation of scores across genders, model outputs were converted using a gender-specific piecewise linear function with 2 segments, such that model outputs of 0 and 100 were maintained, while the gender threshold values were both converted to 65. Model performance was validated on a hold-out dataset of approximately 600 asthma patients and 500 healthy individuals (Table 6). The same model and threshold values were evaluated on over 600 individuals with COPD, more than 800 with persistent cough, and approximately 100 with interstitial lung disease (ILD). Table 7 gives the performance metrics and 95% confidence intervals across conditions using the model score and the same cut-off values for each condition. Odds Ratios for asthma, cough, and ILD are overlapping with an approximately overall mean of ~4. The Odds Ratio for COPD is markedly higher at ~9, and the CI does not overlap with any of the other conditions. This suggests stronger changes in voice acoustic features for this condition relative to the others. Whether there is a connection to more severe clinical presentation of the patients with COPD is not known, as no other assessments or patient-reported symptoms are available in this dataset. The generalizability of this model, trained to differentiate asthma vs. healthy control, to COPD, ILD and cough, gives confidence that extension to other respiratory conditions may be possible.
